# Correlation Between Improved Mating Efficiency and Weakened Scaffold-Kinase Interaction in the Mating Pheromone Response Pathway Revealed by Interspecies Complementation

**DOI:** 10.3389/fmicb.2022.865829

**Published:** 2022-04-14

**Authors:** Tianfang Shi, Junyuan Zeng, Jungang Zhou, Yao Yu, Hong Lu

**Affiliations:** ^1^State Key Laboratory of Genetic Engineering, School of Life Sciences, Fudan University, Shanghai, China; ^2^Shanghai Engineering Research Center of Industrial Microorganisms, Shanghai, China; ^3^Shanghai Collaborative Innovation Center for Biomanufacturing Technology, Shanghai, China

**Keywords:** *Kluyveromyces*, pheromone pathway, complementation, scaffold protein, Ste5, Ste7

## Abstract

Scaffold protein Ste5 and associated kinases, including Ste11, Ste7, and Fus3, are core components of the mating pheromone pathway, which is required to induce a mating response. Orthologs of these proteins are widely present in fungi, but to which extent one protein can be replaced by its ortholog is less well understood. Here, interspecies complementation was carried out to evaluate the functional homology of Ste5 and associated kinases in *Kluyveromyces lactis*, *K. marxianus*, and *Saccharomyces cerevisiae*. These three species occupy important positions in the evolution of hemiascomycetes. Results indicated that Ste5 and associated kinases in *K. lactis* and *K. marxianus* could be functionally replaced by their orthologs to different extents. However, the extent of sequence identity, either between full-length proteins or between domains, did not necessarily indicate the extent of functional replaceability. For example, Ste5, the most unconserved protein in sequence, achieved the highest average functional replaceability. Notably, swapping Ste5 between *K*. *lactis* and *K*. *marxianus* significantly promoted mating in both species and the weakened interaction between the Ste5 and Ste7 might contribute to this phenotype. Consistently, chimeric Ste5 displaying a higher affinity for Ste7 decreased the mating efficiency, while chimeric Ste5 displaying a lower affinity for Ste7 improved the mating efficiency. Furthermore, the length of a negatively charged segment in the Ste7-binding domain of Ste5 was negatively correlated with the mating efficiency in *K. lactis* and *K. marxianus*. Extending the length of the segment in KlSte5 improved its interaction with Ste7 and that might contribute to the reduced mating efficiency. Our study suggested a novel role of Ste5-Ste7 interaction in the negative regulation of the pheromone pathway. Meanwhile, Ste5 mutants displaying improved mating efficiency facilitated the breeding and selection of *Kluyveromyces* strains for industrial applications.

## Introduction

**FIGURE 1:**
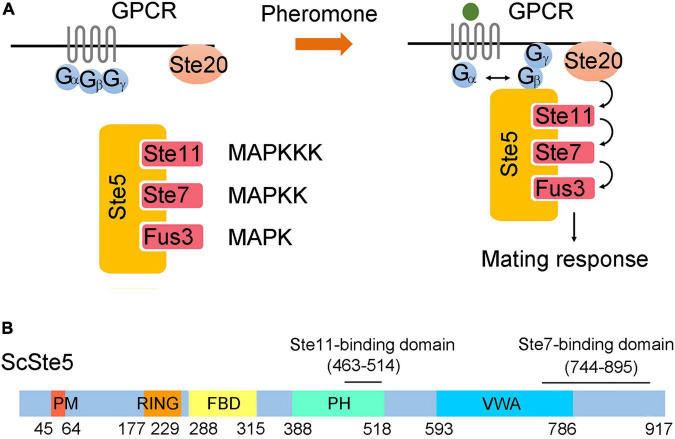
Ste5 and associated kinases in the pheromone pathway. **(A)** Model of the pheromone pathway. Pheromone secreted by haploid cells of different mating types binds to a G protein-coupled receptor and promotes the release of Gβγ dimer from Gα monomer. Ste5 is recruited by Gβγ dimer to the plasma membrane and initiates the activation of a kinase cascade. Activated Fus3 is dissociated from Ste5 and stimulates downstream mating responses. **(B)** Schematic representation of domains of ScSte5. Here and elsewhere in this paper, a prefix of “Sc” stands for protein or gene of *S. cerevisiae*.

In all eukaryotic cells, the mitogen-activated protein kinase (MAPK) cascade plays a pivotal role in triggering and regulating various responses to extracellular stimuli. The mating pheromone response pathway in budding yeast, *Saccharomyces cerevisiae* was among the best models for studying MAPK cascade for more than 30 years (Bardwell, 2005; Alvaro and Thorner, 2016). In this pathway, mating pheromone secreted by haploid cells binds to a G protein-coupled receptor and triggers the dissociation of a G protein heterotrimer (Gαβγ) (Figure 1A). Liberated Gβγ dimer recruits the scaffold protein Ste5 to the plasma membrane (Elion, 2000). Ste5 simultaneously binds three protein kinase, Ste11 (MAPKKK), Ste7 (MAPKK), and Fus3 (MAPK) (Choi et al., 1994). The membrane recruitment of Ste5 facilitates the phosphorylation of Ste11 by the membrane-bound p21-activated protein kinase, Ste20 (Pryciak and Huntress, 1998). Activated Ste11 relays the signal to Ste7 and then Fus3 through sequential phosphorylation (Elion, 2001). Once activated, Fus3 dissociates from Ste5 and phosphorylates a variety of targets to execute the functions required for mating, such as cell-cycle arrest, morphological changes and transcriptional induction (Chang and Herskowitz, 1990; Roberts et al., 2000; Matheos et al., 2004; Yu et al., 2008).

In the pheromone pathway, Ste5 is more than just a scaffold to sequester components of the MAPK cascade. Modular domains of Ste5 play distinct roles in regulating the fidelity and efficiency of signal transduction (Figure 1B; Good et al., 2011). For example, phosphorylation of the PM domain by G1 Cdk blocks membrane binding of Ste5 and thus prevents activation of the mating response at specific stages of the cell cycles (Strickfaden et al., 2007). The VWA domain of Ste5 functions as a co-factor in the allosteric activation of Fus3 by Ste7 (Good et al., 2009). Interaction between Fus3 and the FBD domain of Ste5 promotes autophosphorylation of Fus3, which is required to activate a negative feedback loop that attenuates the pathway output (Bhattacharyya et al., 2006).

The orthologs of Ste5 and associated kinases in the pheromone pathway have been identified throughout the fungal kingdom. However, not much is known about the functional homology of these proteins. Functional homology refers to the extent to which one protein can be functionally replaced by its orthologs. *Kluyveromyces* and *Saccharomyces* occupy interesting positions within the phylogeny of hemiascomycetes. Both genera are part of the “Saccharomyces” complex, a subclade within hemiascomycetes. The Saccharomyces complex is divided by a whole-genome duplication (WGD) event predicted to occur 100 million years ago. The divergence of *Saccharomyces* and *Kluyvermyces* was estimated to be 1.5 × 10^8^years ago, which defines *Saccharomyces* as a post-WGD and *Kluyvermyces* as a pre-WGD species (Wolfe and Shields, 1997). Given the specific position of *Kluyveromyces* and *Saccharomyces* in the evolution of hemiascomycetes, the study of the functional homology of Ste5 and associated kinases from both genera provides valuable information to the relationship between gene sequence and function during evolution.

*Kluyveromyces lactis* and *Kluyveromyces marxianus* are representative species of the *Kluyveromyces* genus (Lachance, 2011). Two species are predicted to separate 10∼25 × 10^6^ years ago (Shen et al., 2018). Both yeasts are famous for their capacity to ferment lactose and thus are frequently found in dairy sources. Besides their traditional applications in the food industry, *K. lactis* and *K. marxianus* have been developed into promising hosts for heterologous protein production and biomass utilization since the 1980s (Fukuhara, 2006; Lane and Morrissey, 2010). However, there were limited studies of the Ste5 and associated kinase in *K. marxianus* and *K. lactis*. Deletion of the gene encoding Ste5 or associated kinase in *K. lactis* caused sterile phenotype (Kawasaki et al., 2008). Orthologs of Ste5 and associated kinases were predicted in *K. marxianus* (Lertwattanasakul et al., 2015), but it is unclear whether they play conserved roles in the mating pheromone response pathway.

In this study, conserved functions of Ste5 and associated kinases in the mating were confirmed in *K. marxianus*. Next, the functional homology of Ste5 and associated kinases was evaluated by the interspecies complementation between *K. lactis*, *K. marxianus* and *S. cerevisiae*. In the assay, the gene was replaced by its ortholog at its natural locus and functional replaceability of the ortholog was measured by a quantitative mating assay. Although Ste5 and associated kinases in *K. lactis* and *K. marxianus* could be functionally replaced by their orthologs, the extent of functional replaceability of the ortholog does not perfectly match the extent of sequence identity. Notably, swapping Ste5 between *K*. *lactis* and *K*. *marxianus* significantly promoted mating and the weakened interaction between the Ste5 and Ste7 might contribute to this phenotype. Furthermore, the length of a negatively charged segment inside the Ste7-binding domain of Ste5 was negatively correlated with the mating efficiency. Our study shed new light on the mechanism of MAPK cascade regulation. Meanwhile, *K. marxianus* and *K. lactis* strains displaying improved mating efficiency might provide the basis for the development of both yeasts for industrial applications.

## Materials and Methods

### Strains and Plasmids

Strains used in this study are listed in Table 1. Plasmids are listed in Supplementary Table 1. Relevant primers are listed in Supplementary Table 2.

**TABLE 1:** Yeast strains.

Name	Description	Sources
FIM-1ΔU	ura3Δ (*K. marxianus*)	Zhou et al., 2018
ATCC8585	*MAT*a (*K. lactis*)	ATCC
S288C	*MAT*α *SUC2 gal2 mal2 mel flo1 flo8-1 hap1 ho bio1 bio6* (*S. cerevisiae*)	Lab stock
AH109	*MAT*a *trp1-901 leu2-3*,*112 ura3-52 his3-200 gal4*Δ *gal80*Δ *LYS2*::*GAL1_*UAS*_-GAL1_*TATA*_-HIS3 GAL2_*UAS*_-GAL2_*TATA*_-ADE2 URA3*::*MEL1_*UAS*_-MEL1_*TATA*_-lacZ* (*S. cerevisiae*)	Clontech
Y187	*MAT*α *ura3-52 his3-200 ade2-101 trp1-901 leu2-3*,*112 gal4*Δ *met*^–^ *gal80*Δ *URA3*::*GAL1_*UAS*_-GAL1_*TATA*_-lacZ* (*S. cerevisiae*)	Clontech
LHP506^a^	*MAT*a *ura3*Δ *HML*Δ	This study
LHP507^a^	*MAT*α *ura3*Δ *HMR*Δ	This study
LHP508^a^	*MAT*a *ura3*Δ *HML*Δ *trp1*Δ	This study
LHP560^a^	*MAT*α *ura3*Δ *HMR*Δ *his3*Δ	This study
LHP561^a^	*MAT*a *ura3*Δ *HML*Δ *trp1*ΔΔ*ste11*Δ	This study
LHP697^a^	*MAT*a *ura3*Δ *HML*Δ *trp1*Δ*Δste7*Δ	This study
LHP698^a^	*MAT*a *ura3*Δ *HML*Δ *trp1*Δ*Δfus3*Δ	This study
LHP563^a^	*MAT*a *ura3*Δ *HML*Δ *trp1*Δ*Δste5*Δ	This study
LHP562^a^	*MAT*a *ura3*Δ *HML*Δ *trp1*Δ*Δste11*Δ::*KlSTE11*	This study
LHP565^a^	*MAT*a *ura3*Δ *HML*Δ *trp1*Δ*Δste7*Δ::*KlSTE7*	This study
LHP566^a^	*MAT*a *ura3*Δ *HML*Δ *trp1*Δ*Δfus3*Δ::*KlFUS3*	This study
LHP564^a^	*MAT*a *ura3*Δ *HML*Δ *trp1*Δ*Δste5*Δ::*KlSTE5*	This study
LHP699^a^	*MAT*a *ura3*Δ *HML*Δ *trp1*Δ*Δste11*Δ::*ScSTE11*	This study
LHP700^a^	*MAT*a *ura3*Δ *HML*Δ *trp1*Δ*Δste7*Δ::*ScSTE7*	This study
LHP701^a^	*MAT*a *ura3*Δ *HML*Δ *trp1*Δ*Δfus3*Δ::*ScFUS3*	This study
LHP702^a^	*MAT*a *ura3*Δ *HML*Δ *trp1*Δ*Δste5*Δ::*ScSTE5*	This study
LHP703^a^	*MAT*a *ura3*Δ *HML*Δ *trp1*Δ*Δste5*Δ::*KlSTE5-Km*BD	This study
LHP704^a^	*MAT*a *ura3*Δ *HML*Δ *trp1*Δ*Δste5*Δ::*KmSTE5-Kl*BD	This study
LHP1043^a^	*MAT*a *ura3*Δ *HML*Δ *trp1*Δ*Δste5*Δ::*KmSTE5*Δ*S*	This study
LHP1044^a^	*MAT*a *ura3*Δ *HML*Δ *trp1*Δ*Δste5*Δ::*KlSTE5-ES*	This study
LHP1045^a^	*MAT*a *ura3*Δ *HML*Δ *trp1*Δ*Δste5*Δ::*ScSTE5*Δ*S*	This study
LHP567^b^	*MAT*a *ura3*Δ	This study
LHP568^b^	*MAT*a *ura3*Δ *HML*Δ	This study
LHP569^b^	*MAT*α *ura3*Δ *HMR*Δ	This study
LHP570^b^	*MAT*a *ura3*Δ *HML*Δ *trp1*Δ	This study
LHP571^b^	*MAT*α *ura3*Δ *HMR*Δ *his3*Δ	This study
LHP572^b^	*MAT*a *ura3*Δ *HML*Δ *trp1*Δ*Δste11*Δ::*KmSTE11*	This study
LHP705^b^	*MAT*a *ura3*Δ *HML*Δ *trp1*Δ*Δste7*Δ::*KmSTE7*	This study
LHP706^b^	*MAT*a *ura3*Δ *HML*Δ *trp1*Δ*Δfus3*Δ::*KmFUS3*	This study
LHP707^b^	*MAT*a *ura3*Δ *HML*Δ *trp1*Δ*Δste5*Δ::*KmSTE5*	This study
LHP708^b^	*MAT*a *ura3*Δ *HML*Δ *trp1*Δ*Δste11*Δ::*ScSTE11*	This study
LHP709^b^	*MAT*a *ura3*Δ *HML*Δ *trp1*Δ*Δste7*Δ::*ScSTE7*	This study
LHP710^b^	*MAT*a *ura3*Δ *HML*Δ *trp1*Δ*Δfus3*Δ::*ScFUS3*	This study
LHP711^b^	*MAT*a *ura3*Δ *HML*Δ *trp1*Δ*Δste5*Δ::*ScSTE5*	This study
LHP712^b^	*MAT*a *ura3*Δ *HML*Δ *trp1*Δ *ste5*Δ::*KlSTE5-Km*BD	This study
LHP713^b^	*MAT*a *ura3*Δ *HML*Δ *trp1*Δ *ste5*Δ::*KmSTE5-Kl*BD	This study
LHP1046^b^	*MAT*a *ura3*Δ *HML*Δ *trp1*Δ *ste5*Δ::*KmSTE5*Δ*S*	This study
LHP1047^b^	*MAT*a *ura3*Δ *HML*Δ *trp1*Δ *ste5*Δ::*KlSTE5-ES*	This study
LHP1048^b^	*MAT*a *ura3*Δ *HML*Δ *trp1*Δ *ste5*Δ::*ScSTE5*Δ*S*	This study

*^a^Isogenic relative to FIM-1ΔU. ^b^Isogenic relative to ATCC8585.*

Deletion or replacement of a gene in *K. marxianus* and *K. lactis* was performed by homologous recombination with the aid of a CRISPR plasmid. Three CRISPR vectors, LHZ296, LHZ301, and LHZ531, were used as backbones to build CRISPR plasmids (Shi et al., 2021). Primers containing 20 bp target sequence were annealed in pairs and inserted into *Sap*I or *Aar*I sites of LHZ296, LHZ301, and LHZ531. Details of resultant CRISPR plasmids were listed in Supplementary Table 1.

In *K. lactis* and *K. marxianus*, *HMR* locus contained a silenced copy of genes determining mating type a (*a1*, *a2*), while *HML* locus contained that of genes determining mating type α (*α1*, *α2*, and *α3*) (Barsoum et al., 2010; Lertwattanasakul et al., 2015). *HMR*a and *HML*α serve as donors during the recombinational process that allows a *MAT*a cell to switch to *MAT*a or vice versa. To prevent mating-type switching in *K. marxianus*, the *HMR* locus was removed. A fragment upstream of *a1* was amplified by the primer pair STF23F/STF23R and a fragment upstream of *a2* was amplified by STF24F/STF24R. Both fragments were ligated and inserted into pMD18-T (Takara, D101A) to obtain LHZ329. Donor sequence was amplified from LHZ329 by STF23F/STF24R and co-transformed with LHZ328 into FIM-1ΔU (*MAT*α) by a lithium acetate-mediated method (Antunes et al., 2000). The resultant strain was named LHP507. Similarly, the *HML* locus was removed from FIM-1ΔU (*MAT*a) to obtain LHP506. To prevent mating-type switching in *K. lactis*, the *HML* locus was removed from LHP567 to obtain LHP568. The *HMR* locus was removed from LHP567 to obtain an intermediate strain (*MAT*a, *ura3*Δ, *HMR*Δ). Then, a CRISPR plasmid LHZ567 was transformed into the intermediate strain. LHZ567 caused DNA double-strand breaks upstream of *a1* and *a2* in the *MAT*a locus, respectively. The *HML*α locus in the intermediate strain provided a template to repair the double-strand breaks and that led to the switch of mating type from *MAT*a to *MAT*α. The resultant strain was named LHP569.

To delete *KmTRP1* in LHP506 for the selection of diploid cells, *KmTRP1* with the flanking sequence was amplified by primer pair STF40F/STF40R. The fragment was ligated with pMD18-T. The ORF of *KmTRP1* in the resultant plasmid was removed by mutagenesis PCR using primer pair STF41F/STF41R to obtain LHZ539. The donor sequence was amplified from LHZ539 by STF40F/STF40R and co-transformed with LHZ538 into LHP506 to obtain LHP508. Similarly, *KmHIS3* was deleted in LHP507 to obtain LHP560. *KmSTE11*, *KmSTE7*, *KmFUS3*, or *KmSTE5* was deleted in LHP508 to obtain LHP561, LHP697, LHP698, and LHP563 respectively. *KlURA3* was deleted in ATCC8585 to obtain LHP567. *KlTRP1* was deleted in LHP568 to obtain LHP570. *KlHIS3* was deleted in LHP569 to obtain LHP571. The 697-705 aa of *KmSTE5* was deleted in LHP508 to obtain LHP1043.

To replace *KmSTE11* with *KlSTE11*, *KmSTE11* with the flanking sequence was amplified by the primer pair STF54F/STF54R and ligated with pMD18-T. A linear fragment was amplified from the resultant plasmid by STF81F/STF81R. The fragment was ligated with the ORF of *KlSTE11* amplified by STF55F/STF55R through Gibson assembly to obtain LHZ544. In LHZ544, the ORF of *KISTE11* was flanked by the natural promoter and terminator of *KmSTE11*. The donor sequence was amplified from LHZ544 by STF54F/STF54R and co-transformed with LHZ542 into LHP508 to obtain LHP562. Similarly, *KmSTE7*, *KmFUS3* or *KmSTE5* in LHP508 was replaced by its ortholog in *K. lactis* to obtain LHP565, LHP566 and LHP564 respectively. *KmSTE11*, *KmSTE7*, *KmFus3* or *KmSTE5* in LHP508 was replaced by its ortholog in *S. cerevisiae* to obtain LHP699∼LHP702. *KlSTE11*, *KlSTE7*, *KlFUS3* or *KlSTE5* in LHP570 was replaced by its ortholog in *K. marxianus* to obtain LHP572, LHP705∼LHP707 respectively, and replaced by its ortholog in *S. cerevisiae* to obtain LHP708∼LHP711. *KmSTE5* in LHP508 was replaced by *KlSTE5-KmBD*, *KmSTE5-KlBD*, *KlSte5-ES* and *ScSTE5*Δ*S* to obtain LHP703, LHP704, LHP1044 and LHP1045 respectively. *KlSTE5* in LHP570 was replaced by *KlSTE5-KmBD*, *KmSTE5-KlBD*, *KmSte5*Δ*S*, and *ScSTE5*Δ*S* to obtain LHP712, LHP713, LHP1046, and LHP1048. *KmSTE5* in LHP707 was replaced by *KlSte5-ES* to obtain LHP1047.

To measure the strength of the interaction between Ste5 and kinases, a series of the pGADT7-Ste5 plasmids and pGBKT7-Ste7 plasmids were constructed. Sequences encoding *KmSTE5*, *KlSTE5*, *ScSTE5*, *KlSTE5-KmBD*, *KmSTE5-KlBD*, *KmSTE5*Δ*S*, *KlSTE5-ES*, and *ScSTE5*Δ*S* was amplified and cloned into *Eco*RI site of pGADT7 (Clontech, 630442) by Gibson assembly. ORFs of Ste7 orthologs were amplified and cloned into *Eco*RI site of pGBKT7 (Clontech, 630448) separately by Gibson assembly.

### Media

*Kluyveromyces marxianus*, *K. lactis*, and *S. cerevisiae* cells were grown in YPD medium (2% peptone, 1% yeast extract, 2% agar for plates). Synthetic complete (SC) media were prepared as described before (Amberg, 2005). ME plates (2% malt extract,3% agar) were prepared for the mating of *K. lactis* cells (Zonneveld and Steensma, 2003). The concentration of agar in ME plates was adjusted to 5% to optimize the mating of *K. marxianus* in this study.

### Sequences of Full-Length Proteins and Domains

Protein sequences of *ScSTE11* (YLR362W), *ScSTE7* (YDL159W), *ScFUS3* (YBL016W), and *ScSTE5* (YDR103W) were downloaded from the Saccharomyces Genome Database (SGD). Protein sequences of *KmSTE11* (KLMA_30080), *KmSTE7* (KLMA_70246), *KmFUS3* (KLMA_20041), *KmSTE5* (KLMA_20171), *KlSTE11* (KLLA0_B13112g), *KlSTE7* (KLLA0_C16577g), *KlFUS3* (KLLA0_E10539g), and *KlSTE5* (KLLA0_F12023g) were downloaded from the KEGG. Domains of Ste11, Ste7, Fus3, and Ste5 in *K*. *marxianus* and *K*. *lactis* were determined by alignment with their counterparts in *S*. *cerevisiae*. The range of domains were determined as follow: ScSte11 SAM (aa 21–83), ScSte11 RBL (aa 116–236), ScSte11 kinase (aa 415–717) (Yerko et al., 2013); KlSte11 SAM (aa 11–73), KlSte11 RBL (aa 107–228), KlSte11 kinase (aa 439–730); KmSte11 SAM (aa 11–73), KmSte11 RBL (aa 107–228), KmSte11 kinase (aa 445–735); ScSte7 kinase (aa 191–466) (Won et al., 2011); KlSte7 kinase (aa 166–442); KmSte7 kinase (aa 155–431); ScFus3 kinase (aa 13–309); KlFus3 kinase (aa 13–327); KmFus3 kinase (aa 13–322); ScSte5 RING (aa 177–229), ScSte5 PH (aa 388–518), ScSte5 Fus3BD (aa 288–315), ScSte5 VWA (aa 593–786), ScSte5 Ste7BD (aa 744–895) (Good et al., 2011); KlSte5 RING (aa 128–181), KlSte5 PH (aa 331–450), KlSte5 VWA (aa 498–683), KlSte5 Ste7BD (aa 647–746); KmSte5 RING (aa 136–189), KmSte5 PH (aa 345–464), KmSte5 VWA (aa 512–702), and KmSte5 Ste7BD (aa 660–763).

### Quantitative Mating Assay

The quantitative mating assay was performed as described before with modifications to accommodate *Kluyveromyces* species (Miller and Kirchmaier, 2014). LHP560 served as a *MAT*α tester strain for *K. marxianus*, and LHP571 served as a tester strain for *K. lactis*. Fresh cells of the experimental *MAT*a strain and *MAT*α tester strain were grown in YPD liquid medium at 30°C overnight until the optical density at 600 nm (OD_600_) was above 12. Cells were washed and suspended in H_2_O at an OD_600_ of 1. Excess amount of tester cells compared with experimental cells was required in the quantitative mating assay in *S. cerevisiae* (Miller and Kirchmaier, 2014). In this study, the optimal ratio between experimental cells and tester cells in the mating of *K. marxianus* was determined as 1:2 and the same ratio was applied in the mating of *K. lactis*. For *K. marxianus*, 0.75 mL *MAT*a cells and 1.5 mL *MAT*α cells were mixed and loaded into a syringe attached to a Swinnex Filter Holder (Millipore, SX0002500). For *K. lactis*, 0.5 mL *MATa* cells and 1 mL *MAT*α were mixed and loaded. The mixture of cells was collected on a nitrocellulose filter (Whatman, 7184-002). The filter, cells side up, was placed onto a ME plate and incubated at 30°C. Unless indicated, the incubation time for *K. marxianus* cells was 4 h, and that for *K. lactis* cells was 10 h. The filter was then transferred to a 50 mL centrifuge tube containing 4 ml H_2_O. Cells were resuspended thoroughly by the vortex. The cells were serial diluted and plated on YEPD for total colony-forming units and on SC-His-Trp plates to select for diploid cells. The mating efficiency was calculated as the number of diploid cells divided by one-third of the number of total colony-forming units since the experimental *MATa* strain was mixed with *MATa* tester strain in a 1:2 ratio. Three different colonies of an experimental strain were assayed in parallel.

### Statistical Analysis

Two-tailed *t*-tests were performed to determine if there is a significant difference between samples. *P-*values of less than 0.05 were considered statistically different.

### Yeast Two-Hybrid System and Quantitative β-Galactosidase Assay

A Matchmaker GAL4 Two-Hybrid System was applied in this study (Clontech, PT3247-1). pGADT7-Ste5 plasmids were transformed into AH109 separately and selected on SC-Leu plate. pGBKT7-Ste7 plasmids were transformed into Y187 separately and selected on SC-Trp plate. A Trp^+^ transformant was mated with a Leu^+^ transformant on YPD and diploid cells were selected on SC-Leu-Trp plates.

The quantitative β-galactosidase assay was performed as described in the Yeast Protocols Handbook (Clontech, PT3024-1). Diploid cells were grown in liquid culture and lysed by freeze/thaw cycles in liquid nitrogen. Activities in the cell lysate were measured by using ONPG (Solarbio, O8040-1g) as a substrate. 1 unit of β-galactosidase is defined as the amount which hydrolyzes 1 μmol of ONPG to *o*-nitrophenol and D-galactose per min per cell. To remove the autoactivation of pGBKT7-Ste7, the unit measured for a diploid containing a pGBKT7-Ste7 plasmid and a void pGADT7 vector was subtracted from the unit of a diploid containing the same pGBKT7-Ste7 plasmid and a pGADT7-Ste5 plasmid. Three different colonies of one diploid strain were subjected to the assay and activities were measured in duplicates.

### RNA Extraction and qPCR

Fresh *K. marxianus* and *K. lactis* cells were grown in YPD liquid medium overnight until the OD_600_ was above 12. Cells of 300 mL overnight cultures were harvested and frozen at −80°C. RNA was extracted from frozen cells using a Quick-RNA Fungal/Bacterial Miniprep kit (Zymo Research, R2010) and were reverse transcribed using a PrimeScript RT Reagent Kit (Takara, RR037A). The qPCR was performed using TB Green Premix Ex Taq (Takara, RR820A). Primers used in qPCR were listed in Supplementary Table 2.

## Results

### Orthologs of Ste5 and Associated Kinases in *K. marxianus* Play Conserved Roles in the Mating Pheromone Pathway

**FIGURE 2:**
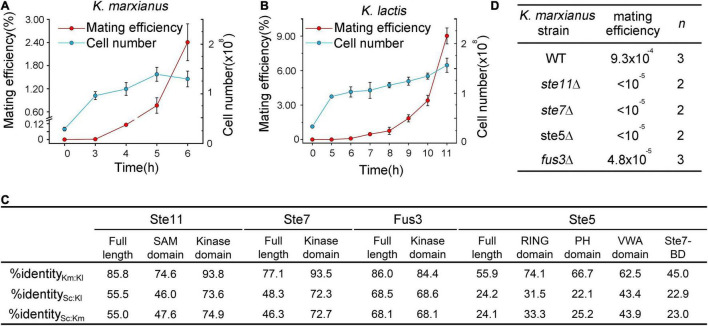
Mating in *K. marxianus* and *K. lactis*. **(A,B)** Time course of mating in *K. marxianus* **(A)** and *K. lactis* **(B)**. Wild-type *K. marxianus* strain LHP508 was mated with tester strain LHP560. Wild-type *K. lactis* strain LHP570 was mated with tester strain LHP571. The quantitative mating assay was performed as described in section “Materials and Methods.” At indicated times, cells were washed from the filters and placed on the mating medium. The total cell number was counted by colonies formed on YPD plates. Mating efficiencies were calculated as described in Methods. The value represented mean ± SD from three biological repeats. **(C)** Identity among orthologs and domains of Ste5 and associated kinases in *S. cerevisiae*, *K. marxianus* and *K. lactis*. Sequences of full-length proteins and domains were obtained as shown in Methods and then aligned. Km was short for *K. marxianus*, Kl for *K. lactis*, Sc for *S. cerevisiae* and BD for binding-domain. **(D)** Mating efficiency of *ste5*Δ, *ste11*Δ, *ste7*Δ and *fus3*Δ mutants in *K. marxianus*. LHP562∼LHP564 was subjected to the quantitative mating assay. The mating efficiency was calculated as the ratio of diploid cells to total cells. “*n*” represents the number of biological repeats.

Same as *S. cerevisiae*, *K. marxianus* and *K. lactis* have two mating types designated as *MAT*a and *MAT*α. In responding to harsh environments, haploid cells of opposite types mate to produce diploid and form spores (Zonneveld and Steensma, 2003; Cernak et al., 2018). In a quantitative mating assay of *K. marxianus*, mixed haploid cells started to form diploid after 3 h, when the number of cells was three times the initial number, the mating efficiency was only 0.093% after 4 h when the number of cells duplicated twice. The efficiency jumped to 0.762% after 5 h (Figure 2A). Similar to *K. marxianus*, *K. lactis* haploid cells started to produce diploid cells after the number of cells was three times the initial number. It took longer (5 h) for *K. lactis* cells to finalize mating, probably because *K. lactis* grows slower than *K. marxianus* (Lane and Morrissey, 2010). When cells were duplicated about two times after 10 hours, the mating efficiency of *K. lactis* was 3.39%, which was much higher than that of *K. marxianus* (Figure 2B). The efficiency climbed to 9% after 11 h. However, *K. lactis* and *K. marxianus* are both poor maters compared to *S. cerevisiae*. Wild-type *S. cerevisiae* cells exhibit mating efficiencies between 15 and 90% (Miller and Kirchmaier, 2014). Different mating behaviors of three species suggested the divergence in regulating mating response, including that in the pheromone pathway.

Based on the sequence identity, orthologs of Ste5 and associated kinases, as well as domains in these proteins, can be recognized in *K. lactis* and *K. marxianus* (Figure 2C). In *K. marxianus*, deletion of genes encoding Ste5, Ste7, Ste11, and Fus3 caused mating defects (Figure 2D). Similar results were reported in *K. lactis* previously (Kawasaki et al., 2008). Therefore, Ste5 and kinases from both *Kluyveromyces* species play similar roles in the mating pheromone pathways as their counterparts in *S. cerevisiae*. The hierarchy of the sequence identities of full-length Ste5 and associated kinases among *K. lactis*, *K. marxianus* and *S. cerevisiae* was determined as follow: Fus3 > Ste11 > Ste7 > Ste5 (Figure 2C). As the least conserved protein, KlSte5 and KmSte5 display similar sequence identities (24%) to ScSte5. KlSte11 and KmSte11, KlSte7 and KmSte7, KlFus3, and KmFus3 also displayed similar sequence identities to their counterparts in *S. cerevisiae*, respectively.

### Ste5 and the Associated Kinases in *K. marxianus* and *K. lactis* Could Be Functionally Replaced by Its Ortholog From Other Species

**FIGURE 3:**
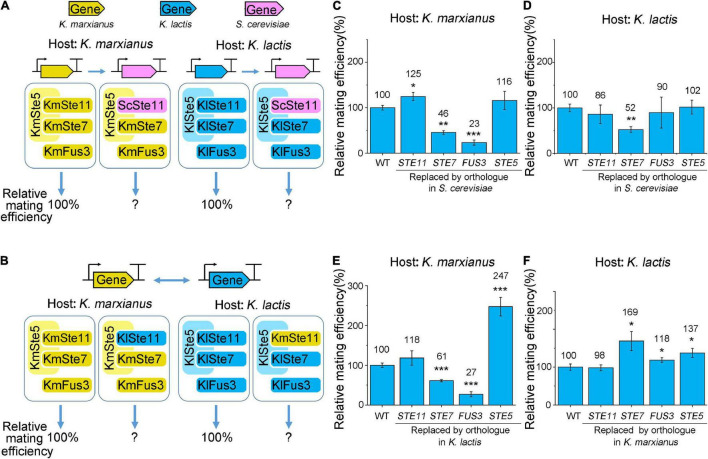
Functional replaceability of orthologs of Ste5 and associated kinases in *K. marxianus* and *K. lactis*. **(A,B)** Schematic representation of the interspecies complementation. Gene encoding Ste5 or kinase in *K. marxianus* or *K. lactis* was replaced by its orthologs in *S. cerevisiae* at the natural locus **(A)** or was swapped between *K. lactis* and *K. marxianus* **(B)**. Here and elsewhere in this paper, a prefix of “Km” stands for *K. marxianus* and “Kl” stands for *K. lactis*. **(C–F)** Relative mating efficiencies of *K. marxianus* and *K. lactis* carrying ortholog of Ste5, Ste7, Ste11 or Fus3. *K. marxianus* strain LHP508 (WT), LHP699∼LHP702 were subjected to the quantitative mating assay in **(C)**. *K. lactis* strain LHP570 (WT), LHP708∼LHP711 were subjected to the assay in **(D)**. LHP508, LHP562, LHP564∼LHP566 were subjected to the assay in **(E)**. LHP570, LHP572, LHP705∼LHP707 were subjected to the assay in **(F)**. The relative mating efficiency of the wild-type strain was designated as 100%. The value represented mean ± SD from three biological repeats. *p*-values were obtained using two-tailed two-sample *t*-tests. (*p < 0.05, **p < 0.01, ***p < 0.001).

To investigate functional homology of Ste5 and associated kinases, the gene encoding Ste5, Ste7, Ste11, or Fus3 in *K. lactis* or *K. marxianus* was replaced by its ortholog in *S. cerevisiae* at its natural locus (Figure 3A), or was swapped between *K. lactis* and *K. marxianus* (Figure 3B). The functional replaceability of an ortholog in the new host was indicated by the mating efficiency of the modified host relative to that of the natural host.

The functional replaceability of ScSte11 and KlSte11 in *K. marxianus* was 125 and 118%, respectively (Figures 3C,E). The functional replaceability of ScSte11 and KmSte11 in *K. lactis* was 86 and 98%, respectively (Figures 3D,F). High functional replaceability of Ste11 orthologs in the interspecies complementation (all above 80%) suggested the function of Ste11 was well conserved in *K. marxianus*, *K. lactis* and *S. cerevisiae*.

Replacing the *STE7* genes in *K. marxianus* and *K. lactis* by their orthologs in *S. cerevisiae* reduced the mating efficiencies to 46 and 52%, respectively (Figures 3C,D). The result suggested the functional replaceability of ScSte7 was reduced to a similar level in *K. marxianus* and *K. lactis*. The functional replaceability of KlSte7 in *K. marxianus* was 61% (Figure 3E), while the functional replaceability of KmSte7 in *K. lactis* was 169% (Figure 3F), suggesting the intrinsic property of KmSte7 in promoting mating was superior to that of KlSte7.

Among Ste5 and three associated kinases, Fus3 was the most conserved protein in terms of the full-length sequence identity. However, the functional replaceability of ScFus3 and KlFus3 in *K. marxianus* was 23 and 27%, respectively, which was among the lowest functional replaceability observed in this study (Figures 3C,E). Therefore, the extent of full-length sequence identity did not perfectly match the extent of functional replaceability. Regarding the identities between domains, the hierarchy of the average identity between the kinase domains in Fus3, Ste11, and Ste7 of *K. lactis*, *K. marxianus*, and *S. cerevisiae* was Ste11 > Ste7 > Fus3 (Figure 2C). The same hierarchy of functional replacement of three kinases was observed in the complementation assay in *K. marxianus* (Figures 3C,E). Therefore, compared to the identities between full-length protein, the identities between kinase domains better matched the extent of functional replaceability of MAPKs. However, there were exceptions. The identity between the kinase domain of KmSte11 and KlSte11 (93.8%) was slightly higher than that of KmSte7 and KlSte7 (93.5%), but the replaceability of KmSte11 in *K. lactis* was only half of that of KmSte7 in *K. lactis* (Figure 3F). Thus, the extent of sequence identity, either between full-length proteins or between domains, did not necessarily indicate the extent of functional replaceability.

Ste5 was the least conserved protein, in terms of full-length protein and domains (Figure 2C). However, Ste5 obtained the highest average functional replaceability in four complementation pairs, supporting the conclusion above. The functional replaceability of ScSte5 in *K. marxianus* and *K. lactis* was 116 and 102%, respectively (Figures 3C,D). Notably, swapping KmSte5 and KlSte5 in *K. marxianus* and *K. lactis* significantly promoted mating efficiency in *K. marxianus* and *K. lactis* to 247 and 137%, respectively (Figures 3E,F). The mechanism underlying this unusual phenotype was investigated afterward.

### Ste5-Ste7 Interaction Strength Was Negatively Correlated With Mating Efficiency in *K. marxianus* and *K. lactis*

Significant improvement of mating efficiency by replacing native protein with its ortholog was not only observed in the cases of Ste5, but also in the replacement of KlSte7 by KmSte7 (Figure 3F). qRT-PCR analysis indicated that mRNA levels of *STE5* or *STE7* orthologs were similar to those of native counterparts (Supplementary Figure 1). It suggested that improved mating efficiency was not due to the change of expression levels.

**FIGURE 4:**
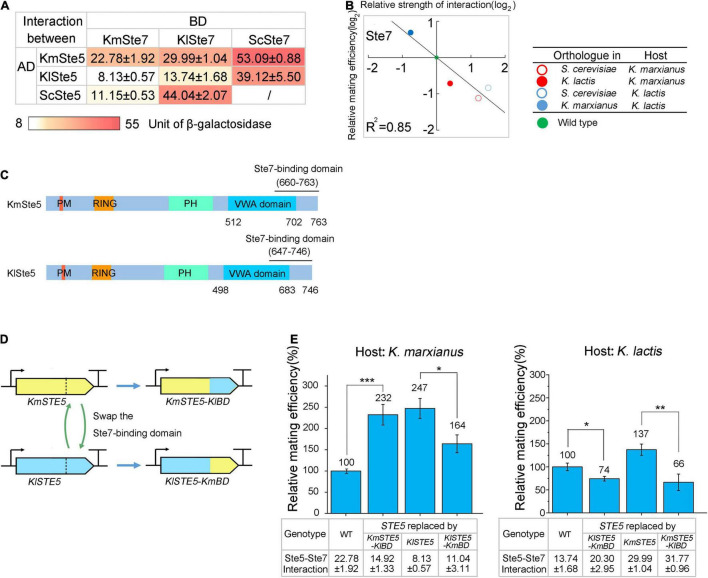
The strength of interaction between Ste5 and Ste7 is negatively correlated with mating efficiency. **(A)** The strength of the interaction between the orthologs of Ste5 and Ste7. In a yeast two-hybrid system, orthologs of Ste5 were fused to Gal4-AD (AD) and those of Ste7 were fused to Gal4-BD (BD). The strength of the interaction between AD-fusion and BD-fusion proteins was indicated by the units of β-galactosidase, encoded by a *lacZ* reporter gene. The strength of the interaction between BD-fusion protein and AD alone was served as the blank and was subtracted. The value represented the mean ± SD from three different colonies. **(B)** The relationship between the relative mating efficiency and the strength of the Ste5-Ste7 interaction. The relative mating efficiency of the strain in which Ste7 was replaced by its ortholog was plotted against the strength of the interaction between host Ste5 and Ste7 ortholog. The binary logarithm of the values was used for plotting. **(C)** Schematic representation of domains of KmSte5 and KlSte5. Domains and potential Ste7-binding regions in KmSte5 and KlSte5 were identified by sequence similarity (Supplementary Figure 2). Counterparts to FBD domain and Ste11-binding region in ScSte5 could not be identified in KmSte5 and KlSte5. **(D)** Schematic representation of swapping Ste7-binding domain between KmSte5 and KlSte5. **(E)** Relative mating efficiency of cells carrying chimeric Ste5. LHP506, LHP507, LHP703, and LHP704 were subjected to the assay of *K*. *marxianus*. LHP570, LHP571, LHP712, and LHP713 were subjected to the assay of *K*. *marxianus*. Value represented mean ± SD from three biological repeats (*p < 0.05, **p < 0.01, ***p < 0.001). The strength of interaction was measured as **(A)** and the value represented the mean ± SD from three different colonies.

The function of a protein is largely determined by its interactions with other proteins. In *S. cerevisiae*, the strongest interaction among Ste5 and associated kinases was between Ste5 and Ste7 (Choi et al., 1994). The change of Ste5-Ste7 interaction might underlie the improved mating efficiency by replacing natural Ste5 or Ste7 with its orthologs. To test this hypothesis, interactions between Ste5 and Ste7 of *K. marxianus*, *K. lactis*, and *S. cerevisiae* was measured by a quantitative β-galactosidase assay in a yeast two-hybrid system (Figure 4A).

When the relative mating efficiencies after replacing natural Ste7 with its ortholog were plotted against the relative strength of the interaction between Ste7 ortholog and natural Ste5, a negative correlation was observed (*R*^2^ = 0.85) (Figure 4B). It suggested that Ste5-Ste7 interaction negatively regulated the mating efficiency in *K. marxianus* and *K. lactis*. In *S. cerevisiae*, the C terminal portion of Ste5 (744–895 aa) was required to interact with Ste7 (Inouye et al., 1997). The Ste7-binding region was recognized in KmSte5 and KlSte5 by sequence similarity (Figure 4C and Supplementary Figure 2). To investigate the relationship between Ste5-Ste7 interaction and mating efficiency, the Ste7-binding region in KmSte5 was replaced by that from KlSte5 and the resultant chimeric protein was named KmSte5-KlBD (BD was short for binding-domain). mRNA level of *KmSTE5-KlBD* was similar to that of *KmSTE5* (Supplementary Figure 1). The strength of the interaction between natural KmSte5 and KmSte7 was 22.78, and that between KmSte5-KlBD and KmSte7 was 14.92. While the relative mating efficiency of wild-type *K. marxianus* cells is 100%, that of the *K. marxianus* cells carrying Ste5-KlBD increased to 232% (Figure 4E). In a reciprocal test, the Ste7-binding region in KlSte5 was replaced by that from KmSte5 to obtain KlSte5-KmBD (Figure 4D). mRNA level of *KlSTE5-KmBD* was similar to that of *KmSTE5* (Supplementary Figure 1). The strength of the interaction between KlSte5 and KmSte7 was 8.13, and that between KlSte5-KmBD and KmSte7 increased to 11.04. The relative mating efficiency of *K. marxianus* cells carrying KlSte5 was 247% and that number reduced to 164% in the cells carrying KlSte5-KmBD (Figure 4E).

In *K. lactis*, KlSte5 was also replaced by two chimeric Ste5, separately. mRNA levels of *KlSTE5-KmBD* and *KmSTE5-KlBD* in *K. lactis* was similar to that of *KlSTE5* (Supplementary Figure 1). The interaction strength between KlSte5 and KlSte7 was 13.74, and that between KlSte5-KmBD and KlSte7 increased to 20.30. The relative mating efficiency of *K. lactis* cells carrying KlSte5-KmBD dropped to 74%, compared to wild-type *K. lactis*. The strength of the interaction between KmSte5 and KlSte7 was 29.99, and that between KmSte5-KlBD and KlSte7 increased to 31.77. The relative mating efficiency of *K. lactis* cells carrying KmSte5 was 137%, and that of cells carrying KmSte5-KlBD reduced to 66% (Figure 4E). The results suggested that, in *K. marxianus* and *K. lactis*, the strengthened interaction between Ste7 and Ste5 attenuated mating, while the weakened interaction improved mating.

### The Length of a Negatively Charged Segment in the Ste7-Binding Domain of Ste5 Was Negatively Correlated With the Mating Efficiency in *Kluyveromyces*

**FIGURE 5:**
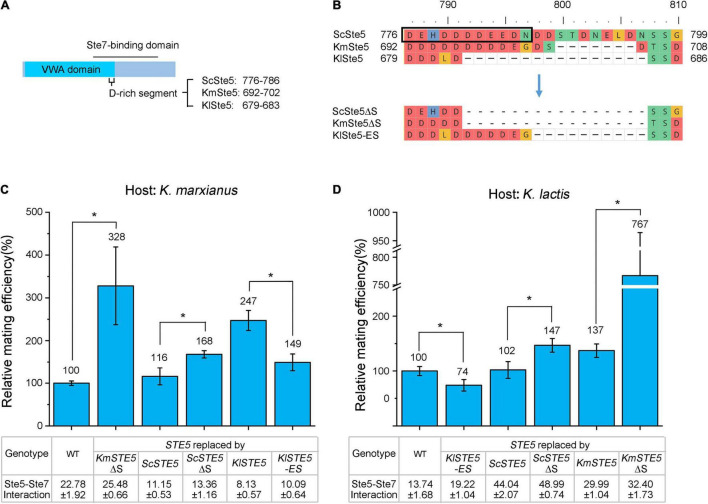
The length of a negatively charged segment in the Ste7-binding domain of Ste5 is negatively correlated with the mating efficiency in *K. marxianus* and *K. lactis*. **(A)** Schematic representation of the position of the negatively charged segment in Ste5. **(B)** Alignment of regions around the negatively charged segment in ScSte5, KmSte5 and KlSte5. The segment in ScSte5 was indicated by a box. The alignment of mutant segments were shown below. **(C,D)** Relative mating efficiency of cells carrying mutant segment. LHP506, LHP507, LHP564, LHP702, LHP1043∼LHP1045 were subjected to the assay of *K*. *marxianus* **(C)**. LHP570, LHP571, LHP707, LHP711, LHP1046∼LHP1048 were subjected to the assay of *K*. *lactis* **(D)**. Value represented mean ± SD from three biological repeats (*p < 0.05). The strength of interaction was measured as Figure 4A and the value represented the mean ± SD from three different colonies.

In *S. cerevisiae*, a Ste5 (V763A, S861P) mutant that was unable to bind Ste7 displayed a clear mating defect, indicating Ste5-Ste7 interaction is required for the positive output of the pheromone pathway (Inouye et al., 1997). This conclusion does not necessarily contradict our assumption that Ste5-Ste7 interaction is negatively correlated with mating efficiency. Ste5 is composed of modular interaction domains, some of which mediate activating or inhibitory regulation with the same binding partner (Good et al., 2011). For example, ScSte5 uses both VWA and FBD domains to interact with ScFus3. The former interaction is required for the allosteric activation of ScFus3 by ScSte7 (Good et al., 2009), while the latter one is believed to attenuate the pathway output (Bhattacharyya et al., 2006). Therefore, Ste5 may use specific motifs inside the Ste7-binding region to mediate inhibitory regulation of the mating pathway. In the Ste7-binding region of ScSte5, a negatively charged segment (DEHDDDDEEDN) was located in the overlapped region of the VWA domain and Ste7-binding domain (Figure 5A). Notably, major differences between the Ste7-binding region of three Ste5 orthologs were around the negatively charged segment (Supplementary Figure 2). In KmSte5, the last asparagine of the segment was changed into a non-polar glycine and seven residues downstream the segment was absent. In KlSte5, half of the segment, as well as the downstream residues were absent (Figure 5B). To investigate the relationship between the segment and improved mating efficiency, the segment of ScSte5 and KmSte5 was reduced to the same length of that of KlSte5 to obtain ScSte5ΔS (S was short for the segment) and KmSte5ΔS, respectively. Meanwhile, the segment of KlSte5 was extended to mimic the segment of KmSte5 and the resultant mutant was named KlSte5-ES (ES was short for the extended segment). mRNA levels of *ScSTE5*Δ*S*, *KmSTE5*Δ*S*, and *KlSTE5-ES* in *K. marxianus* and *K. lactis* were similar to those of native counterparts (Supplementary Figure 1).

The relative mating efficiency of *K. marxianus* cells carrying KmSte5ΔS was 2 times higher than that of cells carrying wild-type Ste5. Similarly, the mating efficiency of *K. marxianus* cells carrying ScSte5ΔS was 44% higher than that of cells carrying ScSte5. Therefore, shortening the negatively charged segment in KmSte5 and ScSte5 improved the mating efficiency in *K. marxianus* (Figure 5C). On the other hand, the relative mating efficiency of *K. marxianus* cells carrying KlSte5-ES was 40% lower than that of cells carrying KlSte5. Therefore, extending the negatively charged segment in KlSte5 reduced the mating efficiency in *K. marxianus* (Figure 5C).

Similar results were observed in *K. lactis*. The relative mating efficiency of *K. lactis* cells carrying ScSte5ΔS was 45% higher than that of cells carrying ScSte5. Notably, the relative mating efficiency of *K. lactis* cells carrying KmSte5ΔS was 4 times higher than that of cells carrying KmSte5 (Figure 5D). Meanwhile, the relative mating efficiency of *K. lactis* cells carrying KlSte5-ES was 26% lower than that of cells carrying wild-type KlSte5 (Figure 5D).

The negatively charged segment composed a binding interface between Ste5 and Ste7. In *S. cerevisiae*, deletion of this region interfered with the ScSte5-ScSte7 interaction and dramatically diminished Ste7-to-Fus3 phosphorylation which was a positive output of the pheromone pathway (Good et al., 2009). Unexpectedly, ScSte5ΔS displayed improved interaction with KmSte7 and KlSte7 (Figures 5C,D). The opposite effects of shortening the segment of ScSte5 on its interaction with Ste7 might be related to the divergence between ScSte7 and KmSte7/KlSte7. Similarly, shortening the segment in KmSte5 also improved its interaction with KmSte7 and KlSte7 (Figures 5C,D), suggesting the function of the segment in ScSte5 and KmSte5 was conserved. Meanwhile, KlSte5-ES displayed improved interaction with KmSte7 and KlSte7 (Figures 5C,D). The results suggested that extending the length of the segment in KlSte5 improved its interaction with Ste7 and that might contribute to the reduced mating efficiency.

In summary, our results suggested that the length of a negatively charged segment in the Ste7-binding domain of Ste5 was negatively correlated with mating efficiency in *Kluyveromyces*. Extending the segment of KlSte5 improved its interaction with KmSte7/KlSte7, while shortening the segment of KmSte5 and ScSte5 also improved their interaction with KmSte7/KlSte7. Thus, the segment of KlSte5, not those of KmSte5 and ScSte5, might mediate the relationship between attenuated Ste5-Ste7 interaction and improved mating.

## Discussion

Orthologous genes descend from a common ancestral DNA sequence and diverge by a speciation event. Orthologous genes are generally assumed to maintain a similar function to that of the ancestral gene (Gabaldon and Koonin, 2013). Divergence of sequence shapes species-specific features of ortholog function (Lynch and Wagner, 2008). Interspecies complementation, in which the natural gene is replaced by its orthologous gene, is one of the best ways to confirm whether an orthologous gene performs equivalent functions (Zill et al., 2010, 2012).

In this study, orthologs of Ste5, Ste7, Ste11, and Fus3 in *K. marxianus* and *K. lactis* were identified by sequence identity. Their function can be replaced by their orthologs in *S. cerevisiae* to different extents. The results suggested the roles of Ste5 and associated kinases in regulating the mating pheromone pathway are conserved in the family Saccharomycetaceae, which includes *Saccharomyces* and *Kluyveromyces* genera (Wolfe et al., 2015). It is very likely that the orthologs from the other genera of the family Saccharomycetaceae clade, such as *Kazachstania*, *Naumovozyma*, *Tetrapisispora*, and *Torulaspora*, are also functionally interchangeable.

However, the extent of sequence identity, either between full-length proteins or between domains, did not necessarily correspond to the extent of functional replaceability. For example, Fus3 was the most conserved protein in full-length but the functional replaceability of KlFus3 and ScFus3 in *K. marxianus* was the lowest. Ste5 was the least conserved protein, in terms of full-length protein and domains, but the average functional replaceability of Ste5 orthologs was the highest.

Usually, a protein can be partially replaced by its ortholog, probably due to the incompatibility between the orthologs and the intracellular environment. For example, the functional replaceability of ScSte11 in *Schizosaccharomyces pombe* was 33%, and that of Spk1 in *S. cerevisiae* was 40% (Styrkarsdottir et al., 1992; Neiman et al., 1993). However, exceptions are not unusual. “Strengthening” mutations can be selected during adaptation (Zill et al., 2010). An ortholog harboring a superior intrinsic property to the natural protein might overcome the incompatibility and produce a better phenotype. For example, replacing ScSir2 or ScSir3 with its orthologs from *Saccharomyces babyanus* improved mating (Zill et al., 2012). In this study, swapping Ste7 between *K. marxianus* and *K. lactis* leads to an improved mating efficiency in *K. lactis* and reduced mating efficiency in *K. marxianus*. The result suggested that the intrinsic property of KmSte7 was superior to that of KlSte7.

Replacing Ste5 in *K. marxianus* by its ortholog from *K. lactis* also improved mating significantly. This phenotype cannot be explained solely by the difference between the intrinsic property of KmSte5 and KlSte5. It was likely that the change of the working environment of Ste5, such as binding partners, also contributed to this phenotype. As shown in Figure 4E, weakened interaction between KlSte5 and KmSte7 was related to the improved mating efficiency in *K. marxianus*. Chimeric Ste5 displaying weakened interaction with Ste7 improved mating, while chimeric Ste5 displaying strengthened interaction with Ste7 reduced mating. The weakened interaction between KlSte5 and KmSte7 might result from the reduced length of the negatively charged segment in the Ste7-binding domain of KlSte5. Consistently, extending the segment in KlSte5 improved its interaction with KmSte7 and reduced mating efficiency (Figure 5C). It was noticeable the segment locates at the overlapped region between VWA and Ste7-binding domain. In *S. cerevisiae*, the VWA domain is required for activation of ScFus3 by ScSte7. Extending the segment in KlSte5 might interfere with Ste7-mediated activation of Fus3. This hypothesis needs further investigation.

In *K. lactis* and *K. marxianus*, reducing the length of the segments in ScSte5 and KmSte5 improved mating (Figures 5C,D), suggesting the full-length segment inhibited mating. However, the inhibition was not mediated by weakening Ste5-Ste7 interaction, because ScSte5ΔS and KmSte5ΔS displayed improved interaction with Ste7. Therefore, other regions in KmSte5 and ScSte5, other than the segment, might mediate the inhibitory effect on mating by attenuating Ste5-Ste7 interaction.

Taking advantage of the combined adaptive traits from parental strains or their tolerance to different stresses, hybrid yeasts were widely utilized in industrial fermentation (Kato et al., 2012; Steensels et al., 2014). Compared with haploids and diploids, polyploidy accelerated evolutionary adaptation (Selmecki et al., 2015), which facilitated the selection of industrial strains. *K*. *marxianus* and *K*. *lactis* were promising hosts for industrial production (Patra et al., 2021). However, the limited mating efficiencies of wild-type *K*. *marxianus* and *K*. *lactis* strains hindered the genetic improvement of both yeasts through constructing hybrid and polyploid strains. In this study, through the engineering of *STE5*, the mating efficiency of *K*. *marxianus* and *K*. *lactis* was elevated by more than two and sixfolds, respectively. The engineered strains will be useful in the breeding of hybrid and polyploid *Kluyveromyces* strains for industrial applications.

In summary, the functional homology of Ste5 and associated kinases in *K. marxianus*, *K. lactis*, and *S. cerevisiae* was evaluated by the interspecies complementation. Results revealed a novel correlation between weakened Ste5-Ste7 interaction and improved mating efficiency. Our study improved the knowledge on classic regulatory circuits underpinning cellular differentiation in yeasts other than *S. cerevisiae*. Meanwhile, our study constructed *Kluyveromyces* strains displaying improved mating efficiency, which facilitated the development of *Kluyveromyces* microbial cell factories.

## Data Availability Statement

The original contributions presented in the study are included in the article/Supplementary Material, further inquiries can be directed to the corresponding author/s.

## Author Contributions

YY and HL designed the study and supervised the project. TS performed most of the experiments and analyzed the data. YY and TS wrote the manuscript. JZe and JZh assisted the construction of strains. All authors have revised the manuscript and approved the final version.

## Conflict of Interest

The authors declare that the research was conducted in the absence of any commercial or financial relationships that could be construed as a potential conflict of interest.

## Publisher’s Note

All claims expressed in this article are solely those of the authors and do not necessarily represent those of their affiliated organizations, or those of the publisher, the editors and the reviewers. Any product that may be evaluated in this article, or claim that may be made by its manufacturer, is not guaranteed or endorsed by the publisher.

## Supplementary Material

Supplementary Table 1 Plasmids used in this study.

Supplementary Table 2 Primers used in this study.
