# Supplementary material for: Correlation Between Improved Mating Efficiency and Weakened Scaffold-Kinase Interaction in the Mating Pheromone Response Pathway Revealed by Interspecies Complementation
Source: Front Microbiol. 2022 Apr 14;13:865829. doi: 10.3389/fmicb.2022.865829 (PMC9048679; doi:10.3389/fmicb.2022.865829)
Supplement: Supplementary file 3 [file Data_Sheet_1.docx]

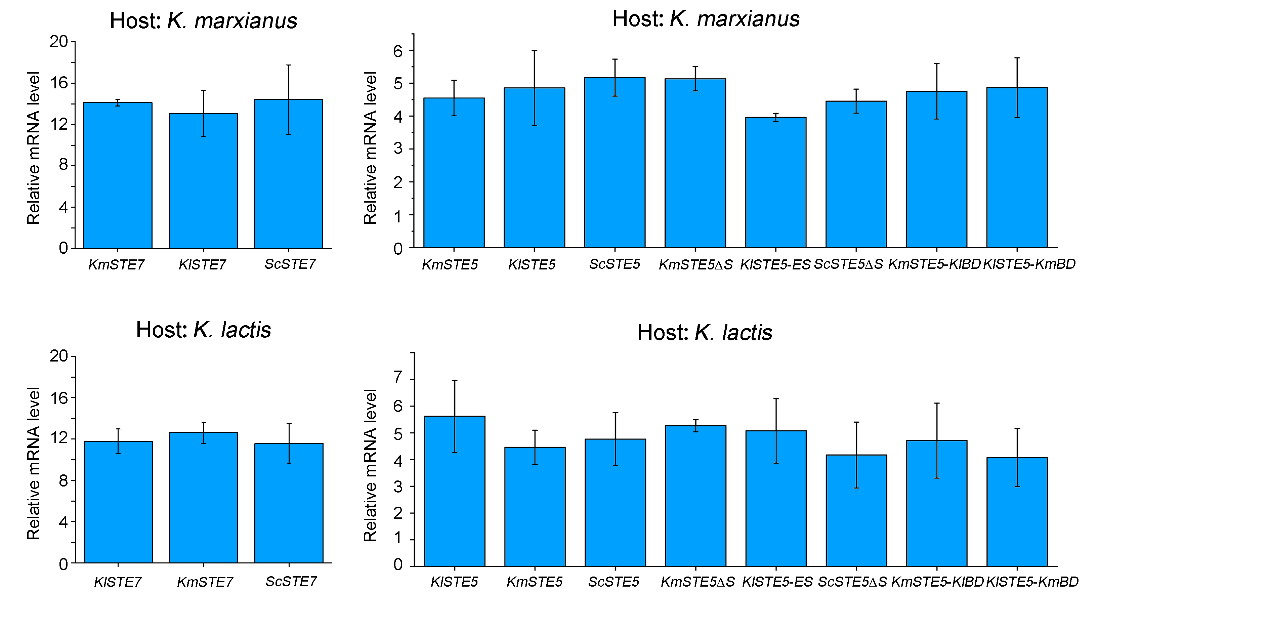


**Figure S1.** Relative mRNA levels of *STE5* and *STE7*. LHP508, LHP564, LHP565, LHP700, LHP702~LHP704, LHP570, LHP705, LHP707, LHP709, LHP711~LHP713, LHP1043~LHP1048 were grown in YPD liquid medium overnight. mRNA levels of wild-type *STE5* and *STE7*, orthologues of *STE5* and *STE7*, and mutant *STE5* and *STE7* were calculated relative to 18s rDNA. The value of 18s rDNA in each sample was designated as 2^18^. Values represented mean ± SD (n = 3).


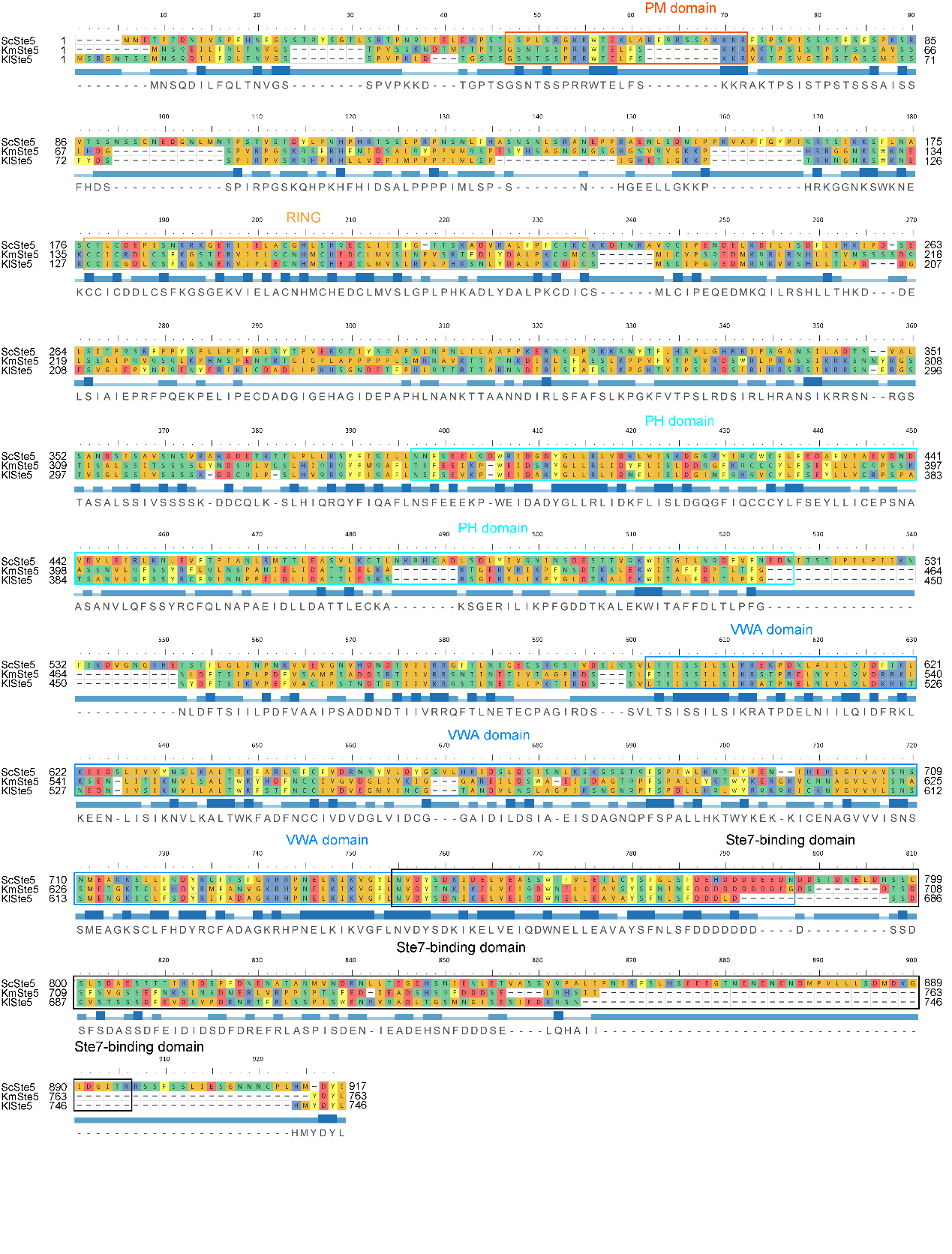


**Figure S2. Alignment of full-length ScSte5, KmSte5 and KlSte5.** The alignment was performed by BioEdit. VWA domain was overlapped with Ste7-binding domain.
